# Supplementary material for: Chromosome evolution and the genetic basis of agronomically important traits in greater yam
Source: Nat Commun. 2022 Apr 14;13:2001. doi: 10.1038/s41467-022-29114-w (PMC9010478; doi:10.1038/s41467-022-29114-w)
Supplement: Supplementary file 3 — Description of Additional Supplementary Files [file 41467_2022_29114_MOESM3_ESM.docx]

## Description of Additional Supplementary Files

#####

##### File Name: Supplementary Data 1

##### Description: Sequencing data.

This Excel file lists the whole-genome shotgun, RNA-seq, and DArT reduced-representation sequencing datasets generated for this work, their metadata, summary statistics, and NCBI SRA deposition information. Data used from external sources for TDa95-310 and TDa95/00328 *D. alata* individuals[^127^](https://paperpile.com/c/spIZH8/ZVyq), *D. rotundata*[^4^](https://paperpile.com/c/spIZH8/TtfA), and *D. dumetorum*[^3^](https://paperpile.com/c/spIZH8/sLLN) are also included.

**File Name: Supplementary Data 2**

##### Description: Delta duplication homoeologous segments.

An Excel file of the homoeologous collinear segments between chromosomes arising from the delta duplication and the positions of the putative centromeric tandem repeat loci.

**File Name: Supplementary Data 3**

##### Description: Relatedness among eight sequenced breeding lines.

An Excel table of pairwise identity-by-descent (IBD) and relatedness coefficients between eight *D. alata* individuals. IBD0, no shared haplotypes; IBD1, one shared haplotype; IBD2, two shared haplotypes; 2*φ*_P_, twice the PREST-plus[^128^](https://paperpile.com/c/spIZH8/lhC40) phi relatedness coefficient, calculated as 0.50·IBD2 + 0.25·IBD1, or the fraction of a diploid genome shared IBD; 2*φ*_K_, twice the phi relatedness coefficient calculated with KING[^32^](https://paperpile.com/c/spIZH8/umIs); PO, parent-offspring; FS, full-sibling; HSFC, half-sibling + first-cousin (non-shared parents are siblings); HS, half-sibling (one shared parent); GG, grandparent-grandchild; AV, avuncular; HAV, half-avuncular; HFC, Half first cousin (sharing only one grandparent); UN, unrelated; 2nd, unspecified second-degree relation; 3rd, unspecified third-degree relation; 4th, unspecified fourth-degree relation.

**File Name: Supplementary Data 4**

##### Description: Species and accessions used in this work.

An Excel table listing nuclear and plastid genome sequences for species used in the phylogenetic and comparative analyses performed in this work. Sequences, where available, are listed by their NCBI GenBank or RefSeq accession numbers.
