# Supplementary material for: Chromosome evolution and the genetic basis of agronomically important traits in greater yam
Source: Nat Commun. 2022 Apr 14;13:2001. doi: 10.1038/s41467-022-29114-w (PMC9010478; doi:10.1038/s41467-022-29114-w)
Supplement: Supplementary file 8 — Reporting Summary [file 41467_2022_29114_MOESM8_ESM.pdf]

nature portfolio

Corresponding author(s): Daniel S. Rokhsar

Last updated by author(s): Jan 19, 2022

Reporting Summary

Nature Portfolio wishes to improve the reproducibility of the work that we publish. This form provides structure for consistency and transparency in reporting. For further information on Nature Portfolio policies, see our Editorial Policies and the Editorial Policy Checklist.

Statistics

For all statistical analyses, confirm that the following items are present in the figure legend, table legend, main text, or Methods section.

n/a	Confirmed	
☐	☒	The exact sample size (*n*) for each experimental group/condition, given as a discrete number and unit of measurement
☐	☒	A statement on whether measurements were taken from distinct samples or whether the same sample was measured repeatedly
☐	☒	The statistical test(s) used AND whether they are one- or two-sided<br>*Only common tests should be described solely by name; describe more complex techniques in the Methods section.*
☒	☐	A description of all covariates tested
☐	☒	A description of any assumptions or corrections, such as tests of normality and adjustment for multiple comparisons
☐	☒	A full description of the statistical parameters including central tendency (e.g. means) or other basic estimates (e.g. regression coefficient) AND variation (e.g. standard deviation) or associated estimates of uncertainty (e.g. confidence intervals)
☐	☒	For null hypothesis testing, the test statistic (e.g. *F*, *t*, *r*) with confidence intervals, effect sizes, degrees of freedom and *P* value noted<br>*Give P values as exact values whenever suitable.*
☒	☐	For Bayesian analysis, information on the choice of priors and Markov chain Monte Carlo settings
☒	☐	For hierarchical and complex designs, identification of the appropriate level for tests and full reporting of outcomes
☐	☒	Estimates of effect sizes (e.g. Cohen's *d*, Pearson's *r*), indicating how they were calculated

Our web collection on statistics for biologists contains articles on many of the points above.

Software and code

Policy information about availability of computer code

Data collection	Leaf Doctor v1.1 (in Supplementary Information)

Data analysis

The following software and their versions are declared in the manuscript Methods sections:
Canu v1.7-221-gb5bffcf, JuiceBox v1.9.0, SSPACE v3, 3D-DNA commit 2796c3b, Arrow v2.2.2 (SMRT Link v6.0.0.47841), FreeBayes v1.1.0-54-g49413aa, VCFtools v0.1.16-16-g954e607, AlphaFamImpute v0.1, JoinMap v4.1, LPmerge v1.7, Guppy v2.3.1, proovread v2.14.1, Pinfish v0.1.0, Minimap2 v2.8, IGC v5.0, PERTRAN v2.4, PASA v2.0.2, EXONERATE v2.4.0, FGENESH+ v3.1.1, FGENESH_EST v2.6, AUGUSTUS v3.3.3, BRAKER1 v1.9, InterProScan v5.17-56.0, BUSCO v3.0.2-11-g1554283, RepeatMasker v4.1.1, RepeatModeler v1.0.11, RepeatModeler v2.0.1, OrthoFinder v2.4.1, ClusterVenn online tool (https://orthovenn2.bioinfotoolkits.net/cluster-venn), BEDtools v2.28.0, R v3.5.3, BLASTP (BLAST+) v2.10.0, DIALIGN-TX v1.0.2, SeqinR v3.6-1, MCscan v1.0.14-0-g58b7710b, PLINK v1.90b6.16, fastq-mcf (ea-utils suite) v1.04.807-18-gbd148d4, BWA-MEM v0.7.17-11-g20d0a13, SAMtools v1.9-93-g0ca96a4, GATK v3.8-1-0-gf15c1c3ef, SnpEff v5.0.c2020-11-25, MAFFT v7.427, Gblocks v0.91b, PhyML v3.3.20190909, IQ-TREE v2.0.3, FigTree v1.4.4, MapTK (v1.4.1-11-g19a5f3a, https://bitbucket.org/rokhsar-lab/gbs-analysis), call-compartments v0.1.2-67-g18fff4a (https://bitbucket.org/bredeson/artisanal), chr-structure.R (v1.0, https://github.com/bredeson/Dioscorea-alata-genomics), run-collinearity.sh (v1.0, https://github.com/bredeson/Dioscorea-alata-genomics), cluster-collinear-bedpe v0.1.2-67-g18fff4a, (https://bitbucket.org/bredeson/artisanal), plot-qtl-gxp.R v1.0 (https://github.com/bredeson/Dioscorea-alata-genomics), plot-qtl-ld.R v1.0 (https://github.com/bredeson/Dioscorea-alata-genomics), IBD v1.0-26-g4cf73ab (https://bitbucket.org/rokhsar-lab/wgs-analysis), snvrate v1.0-26-g4cf73ab (https://bitbucket.org/rokhsar-lab/wgs-analysis)

The more detailed Supplementary Notes sections describe the use of the following additional tools:
ISIS v5.4.7, GIMP v2.8, NxTrim v0.4.2, Jellyfish v2.2.10, Picard v2.16.0, Juicer v1.5.6, BLASR v5.3, FLASH v1.2.11, Juicer Tools v1.8.9, Tandem Repeats Finder v4.09, BLAST v2.2.26, EMBOSS v6.6.0, MUMmer v3.23, IGV v2.9.2, BCFtools v1.9-213-g4411f1e, Python (v2.7.11 and v3.7.6), Minimap2 v2.5-284-g1739a26, ILEC v0.1.3 (https://bitbucket.org/rokhsar-lab/map4cns), allele-balance-filter.py (v1.0-26-g4cf73ab; https://bitbucket.org/rokhsar-lab/wgs-analysis), plot-ibd.R v1.0-26-g4cf73ab (https://bitbucket.org/rokhsar-lab/wgs-analysis)

For manuscripts utilizing custom algorithms or software that are central to the research but not yet described in published literature, software must be made available to editors and reviewers. We strongly encourage code deposition in a community repository (e.g. GitHub). See the Nature Portfolio guidelines for submitting code & software for further information.

Data

Policy information about availability of data

All manuscripts must include a data availability statement. This statement should provide the following information, where applicable:

- Accession codes, unique identifiers, or web links for publicly available datasets
- A description of any restrictions on data availability
- For clinical datasets or third party data, please ensure that the statement adheres to our policy

A reporting summary for this article is available as a Supplementary Information file. Additional data supporting the findings of this work are available throughout the main text, Methods, Supplementary Information, and Supplementary Data. Source Data files are provided with this work. The genome sequence, annotation, and SNP data are browsable at Phytozome (https://phytozome-next.jgi.doe.gov/info/Dalata_v2_1) or YamBase (https://yambase.org/organism/Dioscorea_alata/genome). The D. alata TDa95/00328 nuclear genome (GCA_020875875.1), transcriptome (GJIX00000000.1), plastid (MZ848367.1), and mitochondrion (OK106275.1) assemblies, and Pseudomonas fluorescens chromosome (CP081968.1) were deposited in the NCBI GenBank database. D. rotundata TDr96_F1 and D. dumetorum IboSweet3 plastid sequences were also deposited in the NCBI GenBank database under accessions MZ848368.1 and MZ848369.1, respectively. All sequencing read data generated for this work were deposited in the NCBI Sequence Read Archive (SRA) under BioProject PRJNA666450; see Supplementary Data 1 for individual sample SRA metadata. The genetic linkage maps, phenotype datasets, and DArTseq genotype datasets for all populations, as well as functional annotations for all genes within QTL intervals, were deposited in Dryad (https://doi.org/10.6078/D1DQ54).

Field-specific reporting

Please select the one below that is the best fit for your research. If you are not sure, read the appropriate sections before making your selection.

[x] Life sciences   [ ] Behavioural & social sciences   [ ] Ecological, evolutionary & environmental sciences

For a reference copy of the document with all sections, see nature.com/documents/nr-reporting-summary-flat.pdf

Life sciences study design

All studies must disclose on these points even when the disclosure is negative.

Sample size

Sample size was not calculated prior to experimentation; QTL study sample sizes were instead determined by sample availability, which may be limited by factors such as the flowering rate and crossing efficiency of each population's founders, and the germination and survival rates of their offspring.

For karyotyping a clonally propagated cultivar, variation in count is not expected between cells. It is standard in the field to examine between 2 and 10 metaphase plates with well-spread chromosomes to ensure that no chromosomes were uncounted (due to overlap or loss during preparation). Among the eight micrographs with well-spread chromosomes, all eight supported the same result, 2n = 40 chromosomes.

Data exclusions

No exclusion criteria were pre-established.

All of TDa1419 replicate 3 tuber trait data were excluded from the within-year replicate averaging procedure (see Replication disclosure below), as it was significantly different from replicates 1 and 2 for multiple traits.

Two individual dry matter phenotype measurements were excluded from the TDa1419 population: TDa1419_485, a likely typographical error in data collection, as the fresh weight recorded was less than the recorded weight after drying; and TDa1419_142, which was an extreme

	outlier value. These are now declared in the manuscript Methods section on QTL analysis.
Replication	The five populations grown at IITA were planted as replicates (n = 3), as described in the Supplementary Information. Due to low germination within replicate plots and overlap between plots, not all genotyped samples were able to be phenotyped. Within-year trait replicates for each population were tested for statistically significant differences using the Tukey HSD Test (alpha = 0.05), then averaged using the arithmetic mean.
Randomization	Experimental randomization was not relevant to our study, no behavioral or case-control experiments were performed.
Blinding	Experimental blinding was not relevant to our study, no behavioral or case-control experiments were performed.

Reporting for specific materials, systems and methods

We require information from authors about some types of materials, experimental systems and methods used in many studies. Here, indicate whether each material, system or method listed is relevant to your study. If you are not sure if a list item applies to your research, read the appropriate section before selecting a response.

Materials & experimental systems

n/a	Involved in the study
☒	☐ Antibodies
☒	☐ Eukaryotic cell lines
☒	☐ Palaeontology and archaeology
☒	☐ Animals and other organisms
☒	☐ Human research participants
☒	☐ Clinical data
☒	☐ Dual use research of concern

Methods

n/a	Involved in the study
☒	☐ ChIP-seq
☒	☐ Flow cytometry
☒	☐ MRI-based neuroimaging
